# Coaching by Age: An Analysis of Coaches’ Paternalistic Leadership on Youth Athletes’ Organizational Citizenship Behavior in China

**DOI:** 10.3389/fpsyg.2021.622703

**Published:** 2021-03-23

**Authors:** Juan Li, Sitan Li, Jianbo Hu, Ruichang Chen

**Affiliations:** ^1^School of Economics and Management, Beijing Jiaotong University, Beijing, China; ^2^Moody College of Communication, University of Texas at Austin, Austin, TX, United States; ^3^State Grid Shandong Electric Power Company, Sports Culture Branch, Jinan, China; ^4^Cardiff School of Sport and Health Sciences, Cardiff Metropolitan University, Cardiff, United Kingdom

**Keywords:** social cognitive theory, organizational citizenship behavior, paternalistic leadership, trust in coach, Chinese youth soccer

## Abstract

Based on social cognitive theory, we studied the relationship between coaches’ paternalistic leadership (PL) and youth athletes’ organizational citizenship behavior (OCB) and the mediation effect of athletes’ trust in coaches, in China. This age-specific research was conducted among more than 2,000 Chinese youth soccer players. Overall, 758 youth soccer players, aged 13–18 years, completed a self-report questionnaire. The results showed that the three dimensions of the coaches’ PL have different relationships with OCB, and the differences were due to differences in athletes’ ages. Additionally, we verified the mediation role of trust. Our research conclusions are of great significance to the study of Chinese youth soccer as in-depth research can provide a deeper and more precise understanding of the relationship between PL and the OCB of Chinese youth soccer players. This study expands the literature on social cognitive theory and sheds light on the relationship between coach leadership and athlete OCB by providing extensive evidence.

## Introduction

In the past 30 years, paternalistic leadership (PL) has remained the main field of indigenous leadership research, and research efficiency has increased exponentially in the past 10 years (Pellegrini, 2019). The interest in PL research increased with the review of Pellegrini and Scandura’s (2008) theory (2008) (Pellegrini, 2019). Bedi (2020) recently conducted a meta-analysis on PL, and Jia et al. (2020) conducted a moderated mediation effect study on PL, indicating that the research on PL is in-depth and specific. PL can be defined as a managerial approach that is based on strong discipline and authority combined with fatherly benevolence and moral integrity (Farh and Cheng, 2000; Farh et al., 2014). This leadership style includes three dimensions: authoritative leadership (APL), benevolent leadership (BPL), and moral leadership (MPL) (Farh and Cheng, 2000). Authoritarianism refers to situations in which leaders have absolute authority and control over their subordinates and require their subordinates to obey their orders unconditionally (Farh and Cheng, 2000; Cheng et al., 2004). Benevolence implies that leaders not only focus on the personal well-being of their subordinates but also show concern for the welfare of subordinates’ families (Farh and Cheng, 2000; Cheng et al., 2004). Morality can be broadly described as a leader’s behavior characterized by outstanding personal virtues, self-discipline, and selflessness (Farh and Cheng, 2000; Cheng et al., 2004). Research on PL is often related to the behavioral variables of subordinates (Tang and Naumann, 2015).

The concept of organizational citizenship behavior (OCB) dates to the late 1980s (Organ, 2018). It was defined as spontaneous personal behavior beneficial to the effective operation of an organization undertaken without the direct or explicit approval of the organization’s formal reward system (Organ, 1988). Research on OCB has expanded rapidly, with OCB being applied to various disciplines and different organization types (MacKenzie et al., 2018). Aoyagi et al. (2008) introduced OCB into the sports psychology literature and tested its utility in sports. Research has shown that OCB plays a vital role in sports, improving the operating efficiency and overall performance of sports teams (Wagstaff et al., 2012; Martínez and Tindale, 2015). In sports, the more the team members are engaged in OCB, the more they are invested in the success and well-being of their team (Aoyagi et al., 2008). Furthermore, among adolescents, OCB was found to be conducive to good peer relationships in the group, positive emotional experiences, and personality development (Larson, 2000; He, 2016).

Leadership was found to be an important antecedent of OCB (Podsakoff et al., 2000) and is an essential construct in sports psychology literature. Therefore, Aoyagi et al. (2008) based their initial application of OCB to sports on existing sports psychology theories of leadership and OCB. Their findings indicated that leadership has a significant impact on athletes’ OCB. Most of the research on PL is based on the theory of social exchange (Tang and Naumann, 2015). Due to the particularity of the youth group, we believe that the PL research into this group could benefit from the application of social cognitive theory, similar to the leadership research on youth athletes (Smith and Smoll, 2007).

Social cognitive theory focuses on the interaction between situational and personal factors; that is, individual causes, the environment, and behaviors influence each other in a network of mutual causality (Bandura, 1986). The social cognitive leadership behavior theory model assumes the relationship between situation, cognition, behavior, individual differences, and personality variables (Bandura, 1986). Supportive research on leadership behavior in sports is embodied in a social environment that allows the use of observation techniques to measure public behavior, and an environment that is known to affect the personal and social development of participants (Smith and Smoll, 2007). Research on leadership behavior in the field of sports supports that the sports environment stimulates the psychological participation of coaches and athletes (Smith and Smoll, 2007), improving the possibility of identifying the relationship between leadership behavior and athlete response.

Based on social cognitive theory, Smith and Smoll (2007) believed that leadership is a mediation model because it involves the relationship between sports conditions, coaching behavior and respective athletes’ memory, and athletes’ evaluation and response to coaches (Smoll and Smith, 1984, 1989). The most basic three-element model of coaching influence is as follows: coaching behavior → the athlete’s perception and recall → the athlete’s evaluation response (Smith and Smoll, 2007). This model shows that the coach’s actual behavior has no direct influence on the athlete’s evaluating response (e.g., attitude toward the coach) (Smith and Smoll, 2007). This mediation model requires us to measure the target variables on three different levels: (1) the actual behavior of the coach, (2) how the youth perceive and recall these behaviors, and (3) the youth’s attitude response to the coach (Smith and Smoll, 2007). Using the above theoretical framework, this research explores the relationship among PL, trust in coaches, and youth athletes’ OCB.

From the perspective of social cognition, what is particularly important is the role of situational and individual difference factors in coaching behavior and the reaction of youth athletes to these leadership behaviors (Smith and Smoll, 2007). In complex social and interpersonal relationships, such as sports, individual differences play an important role (Smith and Smoll, 2007). Regarding the PL behavior exhibited by coaches, from the perspective of the cultural roots of the three dimensions of PL theory, the three dimensions are based on traditional Chinese values and concepts, but the cultural power behind these three elements is different, which leads to differences in subordinates’ reactions (Farh and Cheng, 2000). This also explains Tang’s research conclusion from the perspective of social cognition: APL was negatively associated with OCB, while BPL and MPL were positively associated with OCB (Tang and Naumann, 2015). Based on the above analysis, we propose the following hypotheses:

Hypothesis 1: APL will be negatively associated with youth athletes’ OCB.

Hypothesis 2: BPL will be positively associated with youth athletes’ OCB.

Hypothesis 3: MPL will be positively associated with youth athletes’ OCB.

Research in the field of sports has examined the impact of coaches’ PL on athletes (Liou et al., 2007; Chang et al., 2019). Moreover, youth and adult athletes vary in terms of development (Smith and Smoll, 2007). Numerous studies indicate the differences in coaching required for athletes of different ages and competitive levels (Smith and Smoll, 2007; Côté and Gilbert, 2009). From the perspective of PL, when coaching youth and adult athletes, coaches must match their APL, BPL, and MPL behaviors to athletes’ needs to be considered competent age-appropriate coaches. Therefore, we believe that, in China, youth soccer coaches’ PL behaviors should be appropriate to the athletes’ ages. As far as we know, limited studies have focused on coaches’ PL behaviors with youth soccer players in terms of appropriateness regarding athletes’ ages. In this article, we explore the differences in responses to PL among Chinese youth soccer players of different ages. From a developmental point of view, in studies on the youth sports environment, athletes undergo an important period of social and personality development. Coaches not only occupy an important leadership position in the sports environment, but their influence can also be extended to other areas of athletes’ lives (Smith and Smoll, 2007). The way coaches perceive sports conditions, their views on goals, the attitudes and values they convey, and their overall behaviors will obviously affect the psychological development of teenagers (Smith and Smoll, 2007).

From the perspective of youth athletes, the leadership model of social cognitive theory concludes that individual differences in athletes will affect their perception of and responses to coaching behavior (Smith and Smoll, 2007). Studies have proven that there are differences in the perceptions and attitudes of athletes by age, and a factor analysis of coaching behavior identified differences in coaching instructions for athletes aged 8–9, 10–12, and 13–15 years (Smith and Smoll, 2007). Additionally, studies have shown that the older the teenagers are, the higher their need for positive coaching, including positive encouragement, from coaches (Smith and Smoll, 2007). The researchers also suggested that the relationship between age and coaching behavior is worthy of further study (Smith and Smoll, 2007). Moreover, youth Chinese soccer players are at the junior high school stage between the ages of 13 and 15 years, and at the senior high school stage between the ages of 16 and 18 years. With reference to the work of past scholars, we focused on the above two stages to conduct age-specific research. At the same time, in a study on PL and OCB, the scholars argued that the impact of MPL on OCB is different from the impact of BPL and APL on OCB (Farh and Cheng, 2000). Therefore, referring to the differences in subordinates’ responses to the three dimensions in PL and based on social cognitive theory and the above analysis, we propose the following hypotheses for the three dimensions in different age groups:

Hypothesis 1a: APL will be negatively associated with youth athletes’ OCB at ages 13–15.

Hypothesis 1b: APL will be negatively associated with youth athletes’ OCB at ages 16–18.

Hypothesis 1c: The correlation between APL and OCB in the 13–15 age group will be different from that in the 16–18 age group.

Hypothesis 2a: BPL will be positively associated with youth athletes’ OCB at ages 13–15.

Hypothesis 2b: BPL will be positively associated with youth athletes’ OCB at ages 16–18.

Hypothesis 2c: The correlation between BPL and OCB in the 13–15 age group will be different from that in the 16–18 age group.

Hypothesis 3a: MPL will be positively associated with youth athletes’ OCB at ages 13–15.

Hypothesis 3b: MPL will be positively associated with youth athletes’ OCB at ages 16–18.

Hypothesis 3c: The correlation between MPL and OCB in the 13–15 age group will be different from that in the 16–18 age group.

Hypothesis 4: The impact of MPL on OCB will be different from the impact of BPL and APL on OCB.

Trust is a critical issue in sports coaching (Kao et al., 2017) and is defined as the willingness of a party to be vulnerable to the actions of another party based on the expectation that the latter will perform a particular action important to the former, irrespective of their ability to monitor or control the other party (Mayer et al., 1995). Furthermore, trust is often conceptualized as a critical mediation mechanism between leaders and their followers (Dirks, 2000). In this context, Pellegrini and Scandura (2008) concluded that followers who exhibit high levels of trust in their leaders might result in the leaders being more willing to engage in paternalistic practices. Moreover, trust in leaders plays a critical explanatory role in linking PL with the subordinates’ performance (Kao, 2000; Chang and Chi, 2007). Kao et al. (2017) found that the coaches’ capability could improve the athletes’ trust in them. Their research also revealed that in the specific context of constant interaction between coaches and athletes, trust played a mediation role (Kao et al., 2017).

As mentioned above, the leadership model under the framework of social cognitive theory is itself a mediation model (Smith and Smoll, 2007). The mediation variables of this mediation model are the athletes’ feelings and memories, and other mental activities (Smith and Smoll, 2007). The athlete’s trust in the coach is regarded as the athlete’s psychological perception, and it is understandable as a mediation variable in the leadership model under the framework of social cognitive theory. Thus, in this study, we assessed the effect of coaches’ PL on athletes’ OCB and examined the mediation role of trust in the coaches in this relationship. Hence, we propose the following hypotheses:

Hypothesis 5: Trust will mediate the relationship between coaches’ APL and youth athletes’ OCB.

Hypothesis 6: Trust will mediate the relationship between coaches’ BPL and youth athletes’ OCB.

Hypothesis 7: Trust will mediate the relationship between coaches’ MPL and youth athletes’ OCB.

**FIGURE 1:**
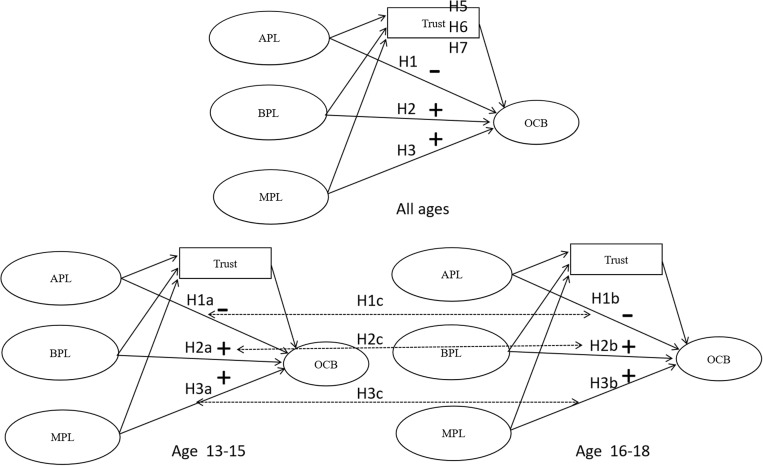
Theoretical research model.

The theoretical research model is presented in Figure 1.

## Materials and Methods

### Participants

This research was reviewed by the Ethics Committee of Beijing Jiaotong University (No. JG201905017) and supported and authorized by the Ethics Committee of Beijing Jiaotong University. With the consent of the guardians, we collected data from China’s 37 Chinese Super League youth teams, 21 Chinese A youth echelon teams, 14 campus soccer teams, and seven social youth soccer teams. The inclusion criterion for the total sample was being between the age of 13–18. The participating teams were widely representative. Approximately 2,055 electronic questionnaires were distributed among 79 youth soccer teams, and 758 were recovered, with a recovery rate of 36.93%. We eliminated six questionnaires of those younger than 13 years old, 11 questionnaires of those older than 19 years old, one questionnaire that did not identify the athlete’s training years, and one questionnaire that was completed in only 63 s and was thus unfinished. Of the remaining 739 valid questionnaires, the questionnaire effective rate was 97.49%, and the average age of the valid questionnaire respondents was 15.30 years old. Among the valid questionnaires, 666 were completed by males with an average age of 15.17 years, and 73 were completed by females with an average age of 16.48 years. Overall, 414 of the respondents were aged 13–15, with an average age of 14.15 years, of whom 403 were males and 11 were females; 325 were from the 16–18 age group, with an average age of 16.76 years, of whom 263 were males and 62 were females. The average length of training of those who effectively participated in the survey was 5.11 years. The data collection period was from July 5 to July 16, 2020, and the average time taken to complete the questionnaire was 481 s.

### Measures

With the author’s consent, we applied the revised coaches’ PL scale (Chen and Kao, 2006), which includes 13 items, including five APL items (e.g., “the coach would ask me to follow his instructions completely in training”), four BPL items (e.g., “the coach’s concern for me would extend to my family or friends”), and four MPL items (e.g., “the coach’s athletic achievement is a model for my learning”). Confirmatory factor analysis results showed that the sample of this scale had acceptable fitness indicators (χ^2^ = 154.762, χ^2^/df = 3.158, CFI = 0.970, CFI = 0.982, TLI = 0.972, SRMR = 0.058, and RMSEA = 0.054). Scale reliability was tested by calculating internal consistency coefficients (Cronbach’s alpha). The accepted reliabilities were 0.88 for APL, 0.84 for BPL, and 0.87 for MPL. Considering the age characteristics of youth athletes, the difficulty of the questionnaire had to be adjusted. Therefore, this study used a five-point Likert-type scale, with 1 indicating total disagreement and 5 indicating total agreement. The scale assessed youth athletes’ perceptions of PL.

This study used a Chinese translation (Dirks and Ferrin, 2000) of the scale of sports trust (Corrales, 2017), which included nine-items (e.g., “most team members trust and respect the coach;” “if I shared my problems with the coach, I know he would respond constructively and caringly;” “the coach approaches his job with professionalism and dedication;” “Given the coach’s past performance, I see no reason to doubt his competence”). This was a one-dimensional measure of the athletes’ trust in their coach. Confirmatory factor analysis results showed that the sample of this scale had acceptable fitness indicators (χ^2^ = 23.52, χ^2^/df = 1.96, GFI = 0.933, CFI = 0.988, TLI = 0.993, SRMR = 0.010, and RMSEA = 0.036). The Chinese version of this scale was consistent with the concept of the original scale and had a Cronbach’s alpha of 0.93. The items were rated using a five-point Likert scale ranging from 1 (“never”) to 5 (“always”).

We chose a revised version of the OCB scale (Podsakoff et al., 1997). This scale consisted of three subscales containing 13 items under the following dimensions: helping (seven items, e.g., “if teammates fall behind in training/make a mistake in the game, we will help each other”), civic virtue (three items, e.g., “in the best interest of the team, I will express my views even if opposed by my teammates and coaching team”), and sportsmanship (three items, e.g., “I always look down on the behavior of other teammates or coaching teams”). To maintain the consistency of the scale, we used a five-point Likert-type scale, ranging from 1 (“never”) to 5 (“always”). Confirmatory factor analysis results showed that the sample of this scale had acceptable fitness indicators (χ^2^ = 124.012, χ^2^/df = 2.584, GFI = 0.974, CFI = 0.987, TLI = 0.980, SRMR = 0.027, and RMSEA = 0.046). The Cronbach’s alpha values of the three subscales were 0.93, 0.70, and 0.62.

### Procedure

The purpose and anonymous nature of the questionnaire were explained to the participants before the survey. Participants voluntarily participated in the survey and did not receive any payment in return for their participation. It was previously indicated that an appropriate study design to measure a mediation effect was to measure the cause before the mediator, and then, the outcome (Podsakoff et al., 2003). In this study, an electronic version of the questionnaire was used. The variables in the questionnaire were arranged in the order of independent, mediator, and dependent variables.

The researchers sent the electronic version of the questionnaire to the youth soccer players through the WeChat group on Friday. During the 2 days off on weekends, athletes could choose any time voluntarily to fill in the questionnaire. Participants used mobile phones or computers to complete the electronic questionnaire. Each person could only fill it out once, and it took about 5–10 min. On Sunday night, the researchers gave a final reminder of the impending deadline of the survey. Coaches and teachers did not intervene, force, or participate in the process of answering the questionnaire.

### Data Analyses

We used SPSSAU and Excel NumXL plug-ins for data analyses. We used the Pearson correlation coefficient to indicate the strength of the relationship, independent variable (APL\BPL\MPL), mediation variable (trust), dependent variable, and control variables (athlete gender, athlete age, athlete training years, coach gender, coach marriage). Finally, we used the Bootstrap sampling inspection method to further verify the mediation effect.

In addition to using structural equations, we performed an analysis to ascertain the difference in coefficients between the two age groups. There are currently three common methods for testing differences in coefficients between groups. First being the “Chow test,” which is tested by introducing cross-multiplication terms. This method assumes that the coefficients of the control variables do not change with the group, and the applicable conditions are the most stringent. Second is the “Seems no correlation” model, “Inspection” allows for differences in the coefficients of the control variables with the sub-sample perturbation terms being related, and the applicable conditions being relatively loose. Finally, the “Fisher combined inspection” method, which is based on the idea of self-sampling, and simulates the overall characteristics through continuous sampling, with the widest application range (Lian and Liao, 2017). The Chow test is currently the most used method (Lian and Liao, 2017), and thus we used it for verification as well.

During the verification process, we also compared the standard regression coefficients. The unstandardized regression coefficient reflects the absolute effect of the change of the independent variable on the dependent variable, while the standardized regression coefficient reflects the relative effect of different independent variables on the dependent variable, which can demonstrate the importance of the influence of different independent variables on the dependent variable (Song, 2007).

## Results

### Descriptive Statistics and Correlations

Descriptive statistics and correlation coefficients for all study variables are reported in Table 1. The data indicated that, except for the lack of correlation between the sportsmanship dimensions of OCB and APL, there was a correlation among the independent, mediation, and dependent variables.

**TABLE 1:** Descriptive statistics of variables.

	Mean	SD	1	2	3	4	5	6	7	8	9	10	11	12
Gender (1)	1.099	0.299	1											
Age (2)	15.302	1.586	0.246**	1										
Training years (3)	5.117	2.079	−0.054	0.337**	1									
Coach gender (4)	1.108	0.311	0.892**	0.214**	−0.093*	1								
Coach marriage (5)	1.283	0.451	0.084*	−0.156**	−0.057	0.129**	1							
APL (6)	3.571	0.937	0.077*	−0.028	−0.117**	0.115**	0.002	1						
BPL (7)	3.881	0.810	0.138**	−0.123**	−0.100**	0.158**	0.008	0.350**	1					
MPL (8)	4.187	0.746	0.125**	−0.148**	−0.145**	0.142**	−0.016	0.231**	0.629**	1				
Trust (9)	4.369	0.611	0.059	−0.140**	−0.121**	0.063	0.014	0.107**	0.537**	0.742**	1			
Help (10)	4.428	0.554	0.041	−0.133**	−0.102**	0.043	0.003	0.142**	0.457**	0.558**	0.721**	1		
CV (11)	4.125	0.650	0.036	−0.118**	−0.071	0.047	−0.013	0.127**	0.456**	0.471**	0.638**	0.701**	1	
SPO (12)	4.220	0.734	0.068	−0.117**	−0.048	0.038	−0.066	0.058	0.278**	0.384**	0.481**	0.482**	0.379**	1

M, mean; SD, standard deviation; APL, authoritative leadership; BPL, benevolent leadership; MPL, moral leadership; CV, civic virtue; SPO, sportsmanship.

*p < 0.05; **p < 0.01.

### Hypothesis Test

According to Wen and Ye (2014) and Fang et al. (2014), the mediation effect test procedure is based on the structural equation model. First, the total effect of coaches’ PL on athletes’ OCB was tested, and we found that the total effect model fitted well (Table 2).

**TABLE 2:** Summary of model goodness of fit index.

	χ^2^/df	GFI	RMSEA	RMR	CFI	NFI	NNFI
Total effect model M1	2.197	0.944	0.040	0.046	0.975	0.956	0.969
APL direct effects model M2	2.034	0.967	0.037	0.037	0.987	0.974	0.982
13–15 APL Direct Effects Model M3	1.549	0.952	0.036	0.037	0.987	0.964	0.983
16–18 APL Direct Effects Model M4	1.724	0.937	0.047	0.048	0.977	0.948	0.970
BPL direct effects model M5	2.003	0.970	0.037	0.023	0.987	0.974	0.982
13–15 BPL direct effects model M6	1.378	0.964	0.030	0.024	0.991	0.968	0.987
16–18 BPL direct effects model M7	1.931	0.934	0.054	0.032	0.972	0.944	0.963
MPL direct effects model M8	5.479	0.909	0.078	0.032	0.943	0.931	0.930
13–15 MPL direct effects model M9	1.818	0.951	0.044	0.024	0.982	0.960	0.975
16–18 MPL direct effects model M10	3.486	0.873	0.087	0.040	0.930	0.905	0.914
Mediating Effect Model M11	1.992	0.934	0.037	0.032	0.977	0.955	0.971
APL Mediation Effect Model M12	1.741	0.958	0.032	0.031	0.987	0.971	0.983
BPL Mediation Effect Model M13	1.866	0.957	0.034	0.022	0.985	0.969	0.980
MPL Mediation Effect Model M14	1.979	0.956	0.036	0.018	0.985	0.970	0.979

*APL, authoritative leadership; BPL, benevolent leadership; MPL, moral leadership; GFI, goodness of fit index; RMSEA, root mean square error of approximation; RMR, root mean square residual; CFI, comparative fit index; NFI, normative fit index; NNFI, non-normed fit index.*

When analyzing the influence of APL on OCB through structural equations (Table 3), this path did not show significance (*z* = −1.397, *p* = 0.162 > 0.05), indicating that APL has no influence on OCB. When analyzing the influence of BPL on OCB, the standardized path coefficient value was 0.255 > 0, and this path showed a significance level of 0.01 (*z* = 3.852, *p* = 0.000 < 0.01); this shows that BPL has a positive impact on OCB. Upon further analysis of the impact of MPL on OCB, the standardized path coefficient value was 0.477 > 0, and this path exhibited a significance level of 0.01 (*z* = 6.752, *p* = 0.000 < 0.01); this indicates that MPL has a positive effect on OCB. Therefore, Hypotheses 2 and 3 were verified, but Hypothesis 1 was not verified.

**TABLE 3:** Summary of model regression coefficients.

*X*	→	*Y*	Non-standardized path coefficient	Standard error	*z*	*p*	Standardized path coefficient
APL	→	OCB	−0.024	0.017	−1.397	0.162	−0.049
BPL	→	OCB	0.212**	0.055	3.852	0.000	0.255
MPL	→	OCB	0.329**	0.049	6.752	0.000	0.477
13–15 APL	→	OCB	−0.024	0.021	−1.17	0.242	−0.056
13–15 BPL	→	OCB	0.061	0.089	0.688	0.492	0.063
13–15 MPL	→	OCB	0.522**	0.082	6.34	0.000	0.616
16–18 APL	→	OCB	−0.028	0.026	−1.057	0.291	−0.055
16–18 BPL	→	OCB	0.227**	0.054	4.198	0.000	0.361
16–18 MPL	→	OCB	0.252**	0.051	4.909	0.000	0.431

**p < 0.01.

*APL, authoritative leadership; BPL, benevolent leadership; MPL, moral leadership; OCB, organizational citizenship behavior.*

Next, we verified by age group. First, in the 13–15-year-old age group (Table 3), the influence path of APL on OCB was not significant (*z* = −1.170, *p* = 0.242 > 0.05), indicating that APL does not have an influence on OCB. The influence path of OCB was also not significant (*z* = 0.688, *p* = 0.492 > 0.05), indicating that BPL has no influence on OCB. The analysis result of MPL’s influence on OCB showed that the standardized path coefficient value was 0.616 > 0, and this path showed a significance level of 0.01 (*z* = 6.340, *p* = 0.000 < 0.01), indicating that MPL has a positive influence on OCB. Therefore, Hypothesis 3a was supported, and Hypotheses 1a and 2a were not verified.

Second, in the 16–18-year-old age group (Table 3), the influence path of APL on OCB was not significant (*z* = −1.057, *p* = 0.291 > 0.05), indicating that APL has no influence on OCB. However, the standardized path coefficient value of the influence of BPL on OCB was 0.361 > 0, and this path exhibited significance at the level of 0.01 (*z* = 4.198, *p* = 0.000 < 0.01). This shows that BPL has a positive impact on OCB. The standardized path coefficient value of MPL’s influence on OCB was 0.431 > 0, and this path showed a significance level of 0.01 (*z* = 4.909, *p* = 0.000 < 0.01), which shows that MPL has a positive influence on OCB. Therefore, Hypotheses 2b and 3b were verified, but Hypothesis 1b was not verified.

The Chow test results of the inter-group difference analysis of the correlation between APL and OCB in the 13–15-year-old age group and the 16–18-year-old age group were (Table 4): Score = 21.667, C.V. = 3.854, and the *p*-value was significant at the 0.05 level. Similarly, the results of the analysis of the differences between BPL and MPL were (Table 4): Score = 12.087, Score = 7.15, C.V. = 3.854, C.V. = 3.854, and the *p*-values of both were significant at the 0.05 level. Hence, in the two age groups of 13–15 and 16–18, the three dimensions of PL, APL\BPL\MPL, had different effects on OCB according to age. This assumes that Hypotheses 1c, 2c, and 3c were all verified.

**TABLE 4:** Chow test results.

Age	Verification item	Score	C.V.	*p*
13–15	APL→OCB	21.667*	3.854	<0.05
16–18				
13–15	BPL→OCB	12.087*	3.854	<0.05
16–18				
13–15	MPL→OCB	7.150*	3.854	<0.05
16–18				
13–15	APL→OCB	29.271*	3.853	<0.05
	BPL→OCB			
13–15	APL→OCB	75.999*	3.853	<0.05
	MPL→OCB			
13–15	BPL→OCB	17.170*	3.853	<0.05
	MPL→OCB			
16–18	APL→OCB	30.697*	3.856	<0.05
	BPL→OCB			
16–18	APL→OCB	52.068*	3.853	<0.05
	MPL→OCB			
16–18	BPL→OCB	12.059*	3.853	<0.05
	MPL→OCB			

*p < 0.05.

*APL, authoritative leadership; BPL, benevolent leadership; MPL, moral leadership; OCB, organizational citizenship behavior.*

We then conducted a more in-depth analysis and performed the Chow test of differences between groups according to the influence of APL\BPL\MPL on OCB in the 13–15-year-old age group. The results of the inter-group differences in the correlation between APL and OCB and the correlation between BPL and OCB were (Table 4): Score = 29.271, C.V. = 3.853, and the *p*-value were significant at the 0.05 level. The results of the differences in the correlation between APL and OCB and the correlation between MPL and OCB were (Table 4): Score = 75.999, C.V. = 3.853, and the *p*-value were significant at the 0.05 level. The results of the differences in the correlation between BPL and OCB and the correlation between MPL and OCB were (Table 4): Score = 17.170, C.V. = 3.853, and the *p*-value were significant at the 0.05 level. Therefore, in the 13–15-year-old age group, the impact of APL\BPL\MPL on OCB was different.

Similarly, in the 16–18-year-old age group, according to the influence of APL\BPL\MPL on OCB, the Chow test of differences between groups was performed. The results of the inter-group differences between the correlation between APL and OCB and the correlation between BPL and OCB were (Table 4): Score = 30.697, C.V. = 3.856, and the *p*-value were significant at the 0.05 level. The results of the difference between the correlation between APL and OCB and the correlation between MPL and OCB were (Table 4): Score = 52.068, C.V. = 3.853, and the *p*-value were significant at the 0.05 level. The results of the difference between the correlation between BPL and OCB and the correlation between MPL and OCB were (Table 4): Score = 12.059, C.V. = 3.853, and the *p*-value were significant at the 0.05 level. In the same way, in the 16–18-year-old age group, the effects of APL\BPL\MPL on OCB were also different.

In the data analysis results of the 13–15-year-old age group (Table 3), the standardized regression coefficient of the impact of MPL on OCB was 0.616>, the standardized regression coefficient of BPL’s impact on OCB was 0.063>, the standardized regression coefficient of APL’s impact on OCB was −0.056 (that is, 0.616 > 0.063 > −0.056). Similarly, in the data analysis results of the 16–18-year-old age group, the standardized regression coefficient of the effect of MPL on OCB was 0.431>, the standardized regression coefficient of BPL’s effect on OCB was 0.361>, the standardized regression coefficient of APL’s effect on OCB was −0.055 (that is, 0.431 > 0.361 > −0.055). So far, Hypothesis 4 has been verified.

After verifying the total effect, we added a mediation variable (trust) to the structural equation model, and all fitting indexes of the model reached an acceptable level (Table 2). The results were as follows (Table 5): when analyzing the influence of APL on Trust, the standardized path coefficient value was −0.080 < 0, and this path was significant at the 0.01 level (*z* = −2.916, *p* = 0.004 < 0.01). This shows that APL has a negative influence on Trust. However, the influence path of BPL on Trust was not significant (*z* = −0.584, *p* = 0.559 > 0.05). This shows that BPL has no influence on Trust. Furthermore, when analyzing the influence of MPL on Trust, the standardized path coefficient value was 0.909 > 0, and this path was significant at the 0.01 level (*z* = 10.710, *p* = 0.000 < 0.01), which shows that MPL has a positive effect on Trust. Finally, upon analyzing the influence of Trust on OCB, the results showed that the standardized path coefficient value was 0.831 > 0, and this path was significant at the 0.01 significance level (*z* = 20.395, *p* = 0.000 < 0.01). This shows that Trust has a positive influence on OCB.

**TABLE 5:** Mediation model path coefficient.

*X*	→	*Y*	Non-standardized path coefficient	Standard error	*z*	*p*	Standardized path coefficient
APL	→	Trust	−0.039**	0.013	−2.916	0.004	−0.080
BPL	→	Trust	−0.033	0.056	−0.584	0.559	−0.039
MPL	→	Trust	0.690**	0.064	10.710	0.000	0.909
Trust	→	OCB	0.808**	0.040	20.395	0.000	0.831

**p < 0.01.

*APL, authoritative leadership; BPL, benevolent leadership; MPL, moral leadership; OCB, organizational citizenship behavior.*

Subsequently, the Bootstrap method results (10,000 times) showed the following (Table 6): for the test of the mediation effect of Trust when APL affects OCB, the 95% confidence interval did not include the number 0 (95% CI: 0.017∼0.141). Therefore, when APL affects OCB, Trust has a mediation role. In other words, APL first affects Trust, and then through Trust it affects OCB. Similarly, when BPL affects OCB, the mediation effect of Trust was tested. The 95% confidence interval did not include the number 0 (95% CI: 0.214∼0.359), which also shows that Trust has a mediation effect when BPL affects OCB. Similarly, BPL first affects Trust, and then through Trust it affects OCB. Again, the same method was used to test the mediation effect of Trust on the impact of MPL on OCB, and the 95% confidence interval did not include the number 0 (95% CI: 0.386∼0.580). This shows that Trust has a mediation effect when MPL affects OCB. In other words, MPL, like BPL and APL, first affects Trust, and then it affects OCB through Trust. So far, Hypotheses 5, 6, and 7 have all been verified.

**TABLE 6:** Bootstrap sampling verification confidence interval.

Item	Effect	Boot SE	BootLLCI	BootULCI
APL⇒T⇒OCB	0.036*	0.016	0.017	0.141
BPL⇒T⇒OCB	0.231**	0.021	0.214	0.359
MPL⇒T⇒OCB	0.373**	0.029	0.386	0.580

*p < 0.05; **p < 0.01.

*APL, authoritative leadership; BPL, benevolent leadership; MPL, moral leadership; OCB, organizational citizenship behavior; T, trust.*

*BootLLCI refers to the lower limit of the 95% interval of Bootstrap sampling, and BootULCI refers to the upper limit of the 95% interval of Bootstrap sampling.*

## Discussion

### Theoretical Implications

Our research examined the relationship between coaches’ PL and athletes’ OCB, and athletes’ trust in coaches in China. We also verified the mediation effect of trust on the relationship between coaches’ PL and the youth soccer players’ OCB. We assumed that the three dimensions of the coaches’ PL have different relationships with OCB, and the differences were due to differences in athletes’ age. We also assumed the mediation effect of trust. Most of the hypotheses were verified.

The conclusion about the relationship between APL, BPL, MPL, and athlete’s OCB is consistent with the findings of previous studies (Tang and Naumann, 2015). For example, Tang and Naumann (2015) concluded that APL has no correlation with OCB, and both BPL and MPL have a positive correlation with OCB. Our empirical evidence is also similar to part of the results of Cheng et al.’s (2004) study on PL and subordinate responses. This shows that there is a certain degree of consistency between the youth group and the adult group (Smith and Smoll, 2007). Next, the conclusion that the correlation between APL, BPL, MPL, and athletes’ OCB differs between groups according to age also conforms to the theoretical background of the triadic reciprocity of the social cognitive theory. In a relevant discussion about the theory, Bandura (1986) proposed the following: “… Many factors are often needed to create a given effect. Because of the multiplicity of interacting influences, the same factor can be a part of different blends of conditions that have different effects. Particular factors are, therefore, associated with effects probabilistically rather than inevitably.” According to this theoretical expression, we can infer that changes in age and coaches’ leadership are factors that change the outcome variable of OCB.

This theory can also explain the inter-group differences in the correlation between APL, BPL, MPL, and athlete’s OCB in the 13–15 age group; that is, APL and BPL are not related to OCB, and MPL is related to OCB. At this age, BPL does not show a correlation with OCB. Based on practical observation experience and the psychological characteristics of adolescents at this age (Smith and Smoll, 2007), we infer that the reason may be that the family and friends of adolescents at this age do not talk to them if they encounter difficulties. There is a lot of communication instead between the coach and players, and the distinguishing feature of BPL is that the coach’s concern for subordinates extends to the family and friends of the subordinates (Farh and Cheng, 2000; Farh et al., 2014), so when the correlation reflects the relationship between BPL and OCB, it will be irrelevant. Another reason may be that teenagers at this age have limited cognitive abilities (He, 2016) and may not be able to fully understand the coach’s BPL behavior. In the same way, social cognitive theory also explains that APL is not related to OCB, but BPL, MPL, and OCB are related in the 16–18 age group.

In the above explanation, the change in the relationship between BPL and OCB in the two age groups of 13–15 and 16–18 from irrelevant to positive, indicates that the relationship between OCB and BPL has become closer than other relationships. This also shows that for youth athletes, similar leadership styles of coaches will have different OCB performances as athletes’ cognitive levels improve. This may be the growth process of youth soccer players. Smith and Smoll (2007) pointed out that as they grow older, youth athletes react differently to coach leadership behavior (Smith and Smoll, 2007). However, they did not conduct an empirical test in their research (Smith and Smoll, 2007), which has been done in our study, the results of which substantiate their point.

In our research, we also found that there are indeed differences in the relationship between APL, BPL, MPL, and OCB in the 13–15 and 16–18 age groups. Moreover, our research found that the impact of MPL on OCB is greater than the impact of APL and BPL on OCB. This conclusion is different from that of Cheng et al. (2004) on adults. In Cheng et al.’s (2004) study, BPL has the greatest impact on the gratitude of subordinates and the repayment of leaders (Cheng et al., 2004). For the compliance of subordinates, it is MPL that has the greatest impact, not the expected APL (Cheng et al., 2004). Cheng et al. (2004) stated that a leadership style that meets the requirements of the times is more effective than a leadership style that does not have an era. We can also state that in youth sports teams, age-compliant leadership is more effective. Our study provides verification of the research conclusions of Cheng et al. (2004), making PL research more in-depth. Our conclusion can also be explained by social cognitive theory and shows that for youth athletes, MPL has a greater impact on OCB. In other words, if we want youth athletes to show more OCB, then the MPL of coaches must be more effective. This result is similar to the conclusion of Bedi’s (2020) recent meta-analysis research.

Additionally, we have verified the mediation role of trust in this study. It is important to note that although there was no correlation between APL and OCB in the overall study of 13–18-year-olds (the correlation was not significant), trust still plays a mediation role. This more fully illustrates the importance of trust (Chen and Kao, 2006) in the relationship between APL, BPL, MPL, and OCB, as trust in other leadership styles (Chang and Chi, 2007). Moreover, this study provides empirical evidence that trust in coaches has a mediation effect on the relationship between APL, BPL, and athletes’ OCB. This extends the findings of Behery et al. (2018) and Xu et al. (2016), who previously studied the mediation effect of trust in leader and justice perceptions, leadership and OCB, and PL and deviant behaviors, respectively, to identify the mediation role of trust.

Based on the above discussion, this study expands the literature on social cognitive theory and sheds light on the relationship between coach leadership and athlete OCB by providing extensive evidence (Aoyagi et al., 2008; Tang and Naumann, 2015).

### Practical Implications

Our research conclusions are of great significance to the study of Chinese youth soccer as in-depth research can provide a deeper and more precise understanding of the relationship between PL and the OCB of Chinese youth soccer players. This research has revealed that as youth people grow up, the same leadership style of different coaches will have different manifestations. This also explains two real-life phenomena: (1) Some coaches are only good at training youth athletes of a certain age; (2) Different coaches of the same youth team bring out different behaviors and sports performances. Thus, in addition to professional factors, we should consider the leadership tendencies of coaches. Should more consideration be given to MPL? Perhaps, the best choice would be to equip teams with fixed coaches according to the ages of athletes so that coaches can exert their maximum efforts in training athletes of that age. At present, the training of youth soccer players in China is still in the learning stage. Thus, more research is still warranted on the management and use of coaches.

### Limitations and Directions for Future Research

There are limitations to this study. First, we used cross-sectional data, which are all self-reported by youth athletes, and no corresponding coaching evaluation scale has been used. We have, however, maximized the sample size to reduce the impact of common method bias. Nevertheless, we can collect data from multiple perspectives in the future. Secondly, OCB has three dimensions. When verifying, we refer to the study of Tang and Naumann (2015) to conduct an overall analysis of the three dimensions of OCB. Although this does not affect our understanding of the impact of PL on OCB, future OCB research can be refined to the specific dimensions of OCB and perform more precise and detailed analysis and demonstrations.

## Data Availability Statement

The raw data supporting the conclusions of this article will be made available by the authors, without undue reservation.

## Ethics Statement

The studies involving human participants were reviewed and approved by Beijing Jiaotong University (JG201905017). The participants and, where necessary, the participants’ legal guardian/next of kin provided written informed consent to participate in this study.

## Author Contributions

JL designed the research and completed the manuscript. JH and RC designed the research with JL and proposed the discussion. SL revised and checked the whole manuscript in the revision process. All authors contributed to the article and approved the submitted version.

## Conflict of Interest

JH was employed by company State Grid Shandong Electric Power Company. The remaining authors declare that the research was conducted in the absence of any commercial or financial relationships that could be construed as a potential conflict of interest.

## References

[B1] Aoyagi M. W. Cox R. H. McGuire R. T. (2008). Organizational citizenship behavior in sport: relationships with leadership, team cohesion, and athlete satisfaction. *J. Appl. Sport Psychol.* 20 25–41. 10.1080/10413200701784858

[B2] Bandura A. (1986). *Social Foundations of Thought and Action: A Social Cognitive Theory.* Englewood Cliffs, NJ: Prentice-Hall.

[B3] Bedi A. (2020). A meta-analytic review of paternalistic leadership. *Appl. Psychol-Int. Rev.* 69 960–1008. 10.1111/apps.12186

[B4] Behery M. Al-Nasser A. D. Jabeen F. El Rawas A. S. (2018). Toxic leadership and organizational citizenship behavior: a mediation effect of followers’ trust and commitment in the Middle East. *Int. J. Bus. Soc.* 19 793–815.

[B5] Chang C. Huang H. C. Huang F. M. Hsieh H. H. (2019). A multilevel analysis of coaches’ paternalistic leadership on burnout in Taiwanese athletes. *Percept. Mot. Skills.* 126 286–304. 10.1177/0031512518819937 30634890

[B6] Chang H. Chi N. (2007). Human resource managers’ role consistency and HR performance indicators: the moderating effect of interpersonal trust in Taiwan. *Int. J. Hum. Resour. Manag.* 18 665–683. 10.1080/09585190601179586

[B7] Chen Y. Kao S. (2006). Research on the reliability and validity evaluation of the sports leadership trust scale. *J. Phys. Educ.* 39 95–102. 10.6222/pej.3903.200609.1108 11670551

[B8] Cheng B. S. Chou L. F. Wu T. Y. Huang M. P. Farh J. L. (2004). Paternalistic leadership and subordinate responses: establishing a leadership model in Chinese organizations. *Asian J. Soc. Psychol.* 7 89–117. 10.1111/j.1467-839X.2004.00137.x

[B9] Corrales E. C. G. (2017). “A baseline study of attention difficulties in teenagers,” in *Academic and Social Inclusion in Colombia*, ed. Taylor J. A. (Bogotá: ÚNICA), 153.

[B10] Côté J. Gilbert W. (2009). An integrative definition of coaching effectiveness and expertise. *Int. J. Sports Sci. Coach.* 4 307–323. 10.1260/174795409789623892 29106335

[B11] Dirks K. Ferrin D. (2000). Trust in leadership: meta-analytic findings and implications for research and practice. *J. Appl. Psychol.* 87 611–628. 10.1037/0021-9010.87.4.611 12184567

[B12] Dirks K. T. (2000). Trust in leadership and team performance: evidence from NCAA basketball. *J. Appl. Psychol.* 85 1004–1012. 10.1037/0021-9010.85.6.1004 11125648

[B13] Fang J. Zhang M. Sun P. (2014). Analysis of multiple mediating effects based on structural equation modeling. *Psychol. Sci.* 37 735–741.

[B14] Farh J. L. Cheng B. S. (2000). “A Cultural Analysis of Paternalistic Leadership in Chinese Organizations,” in *Management and Organizations in the Chinese Context*, eds Li J. T. Tsui A. S. Weldon E. (London, UK: Palgrave), 84–127. 10.1057/9780230511590_5

[B15] Farh J. L. Cheng B. S. Chou L. F. Chu X. P. (2014). “Authority and benevolence: employees’ responses to paternalistic leadership in China,” in *China’s Domestic Private Firms: Multidisciplinary Perspectives on Management and Performance*, eds Tsui A. S. Bian Y. Cheng L. (New York: Taylor and Francis), 230–260.

[B16] He X. (2016). *Adolescent Development and Educational Psychology*, 2nd Edn. Beijing: Higher Education Press.

[B17] Jia J. Zhou S. Zhang L. Jiang X. (2020). Exploring the influence of paternalistic leadership on voice behavior A moderated mediation model. *Empl. Relat.* 42 542–560. 10.1108/ER-06-2019-0263

[B18] Kao S. (2000). *Team culture and coaching leadership: Qualitative and quantitative research approaches.* doctoral dissertation, National Taiwan Normal University, Taipei.

[B19] Kao S. Hsieh M. Lee P. (2017). Coaching competency and trust in coach in sport teams. *Int. J. Sport Sci. Coach.* 12 319–327. 10.1177/1747954117710508

[B20] Larson R. W. (2000). Toward a psychology of positive youth development. *Am. Psychol.* 55:170. 10.1037/0003-066X.55.1.170 11392861

[B21] Lian Y. Liao J. (2017). How to test the coefficient difference between groups after group regression? *J. Zhengzhou Instit. Aeronaut. Ind. Manag.* 35 97–109.

[B22] Liou C. Tsai Y. M. Chen L. H. Kee Y. H. (2007). The influence of paternalistic leadership on athlete burnout. *J. Sport Exerc. Psychol.* 29:S183.

[B23] MacKenzie S. B. Podsakoff N. P. Podsakoff P. M. (2018). “Individual- and Organizational-level Consequences of Organizational Citizenship Behaviors,” in *The Oxford Handbook of Organizational Citizenship Behavior*, eds Podsakoff P. M. MacKenzie S. B. Podsakoff N. P. (Oxford, UK: Oxford University Press), 105–148.

[B24] Martínez R. N. Tindale S. R. (2015). Impact of organizational citizenship behavior on performance in women’s sport teams. *J. Appl. Sport Psychol.* 27 200–215. 10.1080/10413200.2014.978045

[B25] Mayer R. C. Davis J. H. Schoorman F. D. (1995). An integrative model of organizational trust. *Acad. Manag. Rev.* 20 709–734. 10.5465/amr.1995.9508080335

[B26] Organ D. W. (1988). *Issues in Organization and Management Series. Organizational Citizenship Behavior: The Good Soldier Syndrome.* Lexington, MA: Lexington Books/D. C. Health and Company.

[B27] Organ D. W. (2018). “The roots of organizational citizenship behavior,” in *The Oxford Handbook of Organizational Citizenship Behavior*, eds Podsakoff P. M. MacKenzie S. B. Podsakoff N. P. (Oxford, UK: Oxford University Press), 7–18.

[B28] Pellegrini E. K. (2019). *Paternalistic Leadership.* Oxford: Oxford University Press.

[B29] Pellegrini E. K. Scandura T. A. (2008). Paternalistic leadership: a review and agenda for future research. *J. Manage.* 34 566–593. 10.1177/0149206308316063

[B30] Podsakoff P. M. Ahearne M. MacKenzie S. B. (1997). Organizational citizenship behavior and the quantity and quality of work group performance. *J. Appl. Psychol.* 82 262–270. 10.1037/0021-9010.82.2.262 9109284

[B31] Podsakoff P. M. MacKenzie S. B. Lee J. Y. Podsakoff N. P. (2003). Common method biases in behavioral research: a critical review of the literature and recommended remedies. *J. Appl. Psychol.* 88 879–903. 10.1037/0021-9010.88.5.879 14516251

[B32] Podsakoff P. M. MacKenzie S. B. Paine J. B. Bachrach D. G. (2000). Organizational citizenship behaviors: a critical review of the theoretical and empirical literature and suggestions for future research. *J. Manage.* 26 513–563. 10.1016/S0149-2063(00)00047-7

[B33] Smith R. E. Smoll F. L. (2007). “Social-cognitive approaches to coach behaviour,” in *Social Psychology in Sport*, eds Jowett S. Lavallee D. (Champaign, IL: Human Kinetics), 75–90.

[B34] Smoll F. L. Smith R. E. (1984). “Leadership research in youth sports,” in *Psychological Foundations of Sport*, eds Silva J. M. Weinberg R. S. (Champaign, IL: Human Kinetics), 371–386.

[B35] Smoll F. L. Smith R. E. (1989). Leadership behaviors in sport: a theoretical model and research paradigm. *J. Appl. Social Psychol.* 19 1522–1551. 10.1111/j.1559-1816.1989.tb01462.x

[B36] Song N. (2007). *Multivariate Logistic Distribution and its Parameter Estimation.* doctoral dissertation, Beijing University of Technology, Beijing.

[B37] Tang C. Naumann S. E. (2015). Paternalistic leadership, subordinate perceived leader–member exchange and organizational citizenship behavior. *JMO* 21 291–306. 10.1017/jmo.2014.84

[B38] Wagstaff C. Fletcher D. Hanton S. (2012). Positive organizational psychology in sport: an ethnography of organizational functioning in a national sport organization. *J. Appl. Sport Psychol.* 24 26–47. 10.1080/10413200.2011.589423

[B39] Wen Z. Ye B. (2014). Analyses of mediating effects: the development of methods and models. *Adv. Psychol. Sci.* 22 731–745. 10.3724/sp.j.1042.2014.00731

[B40] Xu A. J. Loi R. Ngo H. (2016). Ethical leadership behavior and employee justice perceptions: the mediating role of trust in organization. *J. Bus. Ethics* 134 493–504. 10.1007/s10551-014-2457-4

